# RETRACTION: Effects of Loose Combined Cutting Seton Surgery on Wound Healing and Pain in Patients with High Anal Fistula: A Meta‐Analysis

**DOI:** 10.1111/iwj.70295

**Published:** 2025-03-03

## Abstract

**RETRACTION:** XiJ. , LiW. , LiT. , CaoS. , WeiS.‐C. , XuJ.‐C. , and BiY.‐H. , “Effects of Loose Combined Cutting Seton Surgery on Wound Healing and Pain in Patients with High Anal Fistula: A Meta‐Analysis,” International Wound Journal 21, no. 3 (2024): e14675, 10.1111/iwj.14675.38484699 PMC10940002

The above article, published online on 14 March 2024, in Wiley Online Library (http://onlinelibrary.wiley.com/), has been retracted by agreement between the journal Editor in Chief, Professor Keith Harding; and John Wiley & Sons Ltd. Following an investigation by the publisher, all parties have concluded that this article was accepted solely on the basis of a compromised peer review process. The editors have therefore decided to retract the article. The authors did not respond to our notice regarding the retraction.

**RETRACTION:** J. Xi , W. Li , T. Li , S. Cao , S.‐C. Wei , J.‐C. Xu , and Y.‐H. Bi , “Effects of Loose Combined Cutting Seton Surgery on Wound Healing and Pain in Patients with High Anal Fistula: A Meta‐Analysis,” International Wound Journal 21, no. 3 (2024): e14675, 10.1111/iwj.14675.38484699 PMC10940002

The above article, published online on 14 March 2024, in Wiley Online Library (http://onlinelibrary.wiley.com/), has been retracted by agreement between the journal Editor in Chief, Professor Keith Harding; and John Wiley & Sons Ltd. Following an investigation by the publisher, all parties have concluded that this article was accepted solely on the basis of a compromised peer review process. The editors have therefore decided to retract the article. The authors did not respond to our notice regarding the retraction.

